# Supplementary material for: Modeling glioblastoma heterogeneity as a dynamic network of cell states
Source: Mol Syst Biol. 2021 Sep 16;17(9):e10105. doi: 10.15252/msb.202010105 (PMC8444284; doi:10.15252/msb.202010105)
Supplement: Supplementary file 6 — Source Data for Figure 5 [file MSB-17-e10105-s004.zip › Figure5A_sourcedata/GSEA_3017/nosigdb_stateC.GseaPreranked.1622795002785/DOROSZKO_U3013MG_CLUSTER_2.html]

Details for gene set DOROSZKO\_U3013MG\_CLUSTER\_2[GSEA]

|  || Dataset | state13017 |
| Phenotype | NoPhenotypeAvailable |
| Upregulated in class | na\_pos |
| GeneSet | DOROSZKO\_U3013MG\_CLUSTER\_2 |
| Enrichment Score (ES) | 0.7244778 |
| Normalized Enrichment Score (NES) | 5.091682 |
| Nominal p-value | 0.0 |
| FDR q-value | 0.0 |
| FWER p-Value | 0.0 |
Table: GSEA Results Summary

  

Fig 1: Enrichment plot: DOROSZKO\_U3013MG\_CLUSTER\_2      
 Profile of the Running ES Score & Positions of GeneSet Members on the Rank Ordered List

  

| PROBE | GENE SYMBOL | GENE\_TITLE | RANK IN GENE LIST | RANK METRIC SCORE | RUNNING ES | CORE ENRICHMENT || 1 | ATAD2 |  |  | 7 | 0.649 | 0.0200 | Yes |
| 2 | HIST1H4C |  |  | 9 | 0.603 | 0.0487 | Yes |
| 3 | CENPU |  |  | 10 | 0.588 | 0.0784 | Yes |
| 4 | FAM111A |  |  | 13 | 0.576 | 0.1039 | Yes |
| 5 | EZH2 |  |  | 14 | 0.574 | 0.1329 | Yes |
| 6 | CLSPN |  |  | 17 | 0.561 | 0.1576 | Yes |
| 7 | C21orf58 |  |  | 18 | 0.559 | 0.1859 | Yes |
| 8 | RRM2 |  |  | 19 | 0.542 | 0.2133 | Yes |
| 9 | HMGB2 |  |  | 20 | 0.534 | 0.2403 | Yes |
| 10 | PCLAF |  |  | 29 | 0.484 | 0.2502 | Yes |
| 11 | UBE2T |  |  | 31 | 0.475 | 0.2723 | Yes |
| 12 | GMNN |  |  | 38 | 0.453 | 0.2843 | Yes |
| 13 | ORC6 |  |  | 39 | 0.450 | 0.3070 | Yes |
| 14 | ESCO2 |  |  | 43 | 0.440 | 0.3238 | Yes |
| 15 | RAD51AP1 |  |  | 46 | 0.435 | 0.3422 | Yes |
| 16 | TMPO |  |  | 49 | 0.432 | 0.3604 | Yes |
| 17 | TOP2A |  |  | 52 | 0.426 | 0.3783 | Yes |
| 18 | SPC25 |  |  | 53 | 0.415 | 0.3992 | Yes |
| 19 | BRCA1 |  |  | 54 | 0.410 | 0.4199 | Yes |
| 20 | DHFR |  |  | 55 | 0.405 | 0.4404 | Yes |
| 21 | TYMS |  |  | 60 | 0.390 | 0.4529 | Yes |
| 22 | PHIP |  |  | 61 | 0.387 | 0.4724 | Yes |
| 23 | RRM1 |  |  | 62 | 0.386 | 0.4919 | Yes |
| 24 | SMC2 |  |  | 63 | 0.386 | 0.5114 | Yes |
| 25 | CENPH |  |  | 77 | 0.366 | 0.5062 | Yes |
| 26 | SMC4 |  |  | 78 | 0.365 | 0.5247 | Yes |
| 27 | ITGB3BP |  |  | 79 | 0.364 | 0.5431 | Yes |
| 28 | CDCA5 |  |  | 81 | 0.361 | 0.5595 | Yes |
| 29 | MIS18BP1 |  |  | 89 | 0.355 | 0.5647 | Yes |
| 30 | USP1 |  |  | 92 | 0.352 | 0.5788 | Yes |
| 31 | ATAD5 |  |  | 97 | 0.345 | 0.5889 | Yes |
| 32 | FEN1 |  |  | 98 | 0.342 | 0.6062 | Yes |
| 33 | NUSAP1 |  |  | 100 | 0.340 | 0.6216 | Yes |
| 34 | NCAPD3 |  |  | 103 | 0.334 | 0.6348 | Yes |
| 35 | H2AFX |  |  | 115 | 0.322 | 0.6310 | Yes |
| 36 | HIRIP3 |  |  | 116 | 0.321 | 0.6472 | Yes |
| 37 | PSMC3IP |  |  | 118 | 0.319 | 0.6615 | Yes |
| 38 | CCDC34 |  |  | 120 | 0.318 | 0.6758 | Yes |
| 39 | KIF15 |  |  | 136 | 0.302 | 0.6637 | Yes |
| 40 | NCAPG |  |  | 137 | 0.301 | 0.6789 | Yes |
| 41 | CENPK |  |  | 139 | 0.299 | 0.6921 | Yes |
| 42 | LMNB1 |  |  | 142 | 0.293 | 0.7033 | Yes |
| 43 | ASF1B |  |  | 143 | 0.291 | 0.7180 | Yes |
| 44 | RFC3 |  |  | 156 | 0.283 | 0.7104 | Yes |
| 45 | MYBL2 |  |  | 164 | 0.278 | 0.7117 | Yes |
| 46 | CIP2A |  |  | 170 | 0.272 | 0.7163 | Yes |
| 47 | MAD2L1 |  |  | 174 | 0.270 | 0.7245 | Yes |
| 48 | CDK1 |  |  | 191 | 0.255 | 0.7082 | No |
| 49 | MT2A |  |  | 566 | -0.575 | 0.0547 | No |
Table: GSEA details [plain text format]

  

Fig 2: DOROSZKO\_U3013MG\_CLUSTER\_2: Random ES distribution      
 Gene set null distribution of ES for **DOROSZKO\_U3013MG\_CLUSTER\_2**
